# Perceptions of physical activity and walking in an early stage after stroke or acquired brain injury

**DOI:** 10.1371/journal.pone.0173463

**Published:** 2017-03-08

**Authors:** Karin Törnbom, Katharina S. Sunnerhagen, Anna Danielsson

**Affiliations:** 1 Research group for Rehabilitation Medicine, Institute of Neuroscience and Physiology, Department of Clinical Neuroscience, Sahlgrenska Academy, University of Gothenburg, Sweden; 2 Centre for Person-Centred Care (GPCC), University of Gothenburg, Gothenburg, Sweden; 3 Unit of physiotherapy, Department of Health and Rehabilitation, Institute of Neuroscience and Physiology, Sahlgrenska Academy, University of Gothenburg, Sweden; Fraunhofer Research Institution of Marine Biotechnology, GERMANY

## Abstract

**Background:**

Physical activity has been established as being highly beneficial for health after stroke. There are considerable global efforts to find rehabilitation programs that encourage increased physical activity for persons with stroke. However, many persons with stroke or acquired brain injury do not reach recommended levels of physical activity and increased knowledge about why is needed. We aimed to explore views and experiences of physical activity and walking among persons with stroke or acquired brain injury.

**Method:**

A qualitative study was conducted, among persons with stroke (n = 8) or acquired brain injury (n = 2) from a rehabilitation unit at Sahlgrenska University Hospital in Sweden. Semi-structured in-depth interviews were held about perceptions and experiences of walking and physical activity in general. Data were analyzed using qualitative content analysis, with categories that were determined inductively.

**Results:**

Physical activity in general and walking ability more specifically were considered very important by the participants. However, physical activity was, regardless of exercising habits pre-injury, associated with different kinds of negative feelings and experiences. Commonly reported internal barriers in the current study were; fatigue, fear of falling or getting hurt in traffic, lack of motivation and depression. Reported external barriers were mostly related to walking, for example; bad weather, uneven ground, lack of company or noisy or too busy surroundings.

**Conclusion:**

Persons with stroke or acquired brain injury found it difficult to engage in and sustain an eligible level of physical activity. Understanding individual concerns about motivators and barriers surrounding physical activity may facilitate the work of forming tailor-made rehabilitation for these groups, so that the levels of physical activity and walking can increase.

## Introduction

Each year, 16 million people will have a stroke worldwide, and of them around 5 million are left with disabilities [1]. Disability following stroke is very common and stroke is the leading cause of long term incapacity among the elderly [2]. Persons with stroke are at high risk for recurrent cardiovascular events, with an estimated third of people who have had a stroke having another stroke within 5 years [3].

Physical activity (PA) is defined as any bodily movement produced by skeletal muscles that requires energy expenditure and includes general daily activities [4]. Exercise is a sub category of PA that is planned, regular and aimed at improvement of body functions. In casual language the expressions “physical activity” and “exercise” are used more or less interchangeably and therefore, the term PA will be used solely in the following article.

It is well known that persons with stroke are less active compared to healthy peers, with around half the daily step count and fitness levels well below the average for their age [5, 6]. Low aerobic capacity has been shown after acquired brain injury (ABI) as well [7]. It has also been established that regular PA of at least moderate intensity reduces the risk of stroke [6, 8]. However, achieving sufficient PA levels for persons with stroke or ABI has proven difficult [9]. Understanding why persons with stroke or ABI are less active than their healthy peers is vital if they are to be supported to meet the recommended levels of PA [5, 8]. Already at an early stage, it is crucial that we understand the barriers and motivators to PA after stroke or ABI [10, 11]. If rehabilitation starts early post-stroke, physical functioning improves faster and the chances for maintenance of PA levels are higher [12].

Common consequences of stroke include; deficits in strength, balance, communication, cognition, mobility, and walking [13, 14]. Among these stroke related impairments, the most significant issue cited by persons living with stroke is improving walking ability [15, 16], and accordingly gait recovery is reported as the most common goal for rehabilitation [17].

Qualitative research focuses on individuals’ experiences and is presented as thoughts, feelings, attitudes and perceptions. Thus, its main aim is to develop new knowledge based on participants’ own experiences, rather than a predefined hypothesis [18]. Through qualitative research participants experiences can be collected [18] and interventions that are developed can thereby be evidence based [19].

Considerable global efforts have been made to find evidence-based rehabilitation programs to improve stroke recovery [20]. Despite these efforts, the best way to implement and sustain PA for persons with stroke or ABI remains unclear [8, 21]. To obtain answers for these questions we need to investigate perceptions held by persons with stroke about PA and walking, which is a relatively new, developing and highly important research field [22]. There are existing qualitative studies that aim to understand views about PA, as well as barriers and facilitators after stroke [10, 20, 23–25], but knowledge about experiences of walking post-stroke is scarce [26, 27]. The aim of the current study was to explore views and experiences about PA and walking among persons with stroke or acquired brain injury.

## Methods

### Participants

To be included in the study participants had to; (1) be >18 years of age, (2) have a diagnosis of stroke or acquired brain injury with similar impairments (3) be at the neuro rehabilitation unit at the hospital, (4) have impaired walking ability due to e.g. balance, coordination or endurance problems, based on clinical assessment or observation by the participant’s physical therapist (5) have experience of walking training, and the ability to walk for at least 10 minutes with or without a walking aid. Exclusion criteria were: communicative or cognitive impairment restricting an interview, severe visual or hearing loss, heart disease, dizziness or pain of a severity level restricting walking exercise on a treadmill.

Participants were purposely recruited by the second author (A.D, physical therapist) in order to mirror the actual rehabilitation population in terms of distribution according to age, sex and severity of disability. The second author approached participants face-to-face at the rehabilitation centre, and none of the persons asked refused to participate. For descriptive purposes sex, age, diagnosis, time since onset and use of walking aids were registered. In addition, the comfortable walking speed was measured using a 30 meter walking test [28] and the Fugl-Meyer Assessment of motor function in the lower extremity [29]. Three women and seven men, with a median age of 51 (38–64), enrolled in intensive team rehabilitation between 2 and 10 months after onset of stroke (n = 8) or acquired brain injury (ABI) with similar symptoms (n = 2) who met the criteria were included in the study. As a part of the rehabilitation program participants were encouraged to ambulate outside the hospital, with or without assistance.

### Design and procedure

This study follows the consolidated criteria for reporting qualitative research (COREQ) checklist. Semi-structured individual interviews with open-ended questions were conducted by the first author (K.T.)*, to explore views of physical exercise and walking after stroke [30, 31]. All interviews took place in a quiet and private room at the rehabilitation center.

The interviewer, being in the profession of social sciences and without preconceptions related to the field of physical therapy, was able to approach the interviews with an open mind. That the interviewer was not a part of the rehabilitation team also allowed participants to speak freely without alliance to healthcare providers.

Interviews lasted from 19 to 61 min (mean 36 min), and was audio-recorded. An interview guide (S1 File), based on research questions posed in the study, was used to guide the interviews. The questions were then followed by different probing questions that altered depending on the answers given. Participants were also free to express perceptions and thoughts and as result the contents diverged from the interview guide. Clarifications were used at all times when the interviewer was uncertain about the meaning of what was being discussed. For example; “did I understand you correctly, you walked a lot more before you had the stroke?”.

Each interview began with asking the participants to fill in a questionnaire; the Physical Activity Scale for the Elderly (PASE) [32, 33] reporting their physical activities performed during the last week. Then, they were asked to describe the event of their injury, allowing researchers to obtain the context from which that personal experience and perceptions derived.

### Research ethics

Written informed consent was obtained from all participants prior enrollment. The study was approved by the Regional Ethical Review Board in Gothenburg, registration number 060–12. All interviews have been conducted according to the principles expressed in the Declaration of Helsinki.

### Analysis

The semi structured interviews were analyzed using conventional qualitative content analysis as proposed by Graneheim and Lundman [34]. The qualitative content analysis is a suitable method when the aim is to describe a phenomenon where research literature is limited [35].

At the end of each interview the recorded contents were transcribed verbatim. Interviews were read several times by the two authors to obtain full insight and understanding of the phenomena under study [34]. The abstraction began with searching the text for meaning units, which consisted of sentences related to the same meaning. The process of reducing data was then followed by reducing meaning units into shorter codes. The codes were compared according to similarities and differences and categories were formed. The categories were then analyzed to search for latent, underlying meanings, and themes and subthemes emerged [34, 35]. Hence, all themes derived from the data.

All stages of the analysis were performed through collaboration between the two authors and a process of reflection and discussion was held until consensus was reached at each stage [34]. As the two researchers represented different professions; a social scientist and a physical therapist, different aspects could be brought up.

## Results

Participants’ characteristics are shown in Table 1. The findings are presented through themes and sub-themes presented and illustrated with quotes from participants.

**Table 1:** Participants characteristics and physical activity scores.

Sex	Age (yr)	Diagnosis	Time since onset (months)	Motor function FMA-LE*	Comfortable walking speed (m/s)	Walking aid	PASE score** (%)***
male	53	stroke	6	34	1.28	none	79 (39)
male	45	encephalitis	2	34	1.11	none	28 (19)
male	38	cerebral trauma	5	34	1.56	none	114 (-)
female	41	stroke	7	34	1.36	none	62 (44)
male	64	stroke	3	34	1.36	none	70 (43)
male	51	stroke	6	21	1.07	stick	57 (28)
male	50	stroke	4	20	0.58	stick	-
female	53	stroke	2	34	1.25	none	51 (30)
male	62	stroke	8	32	1.29	none	27 (16)
female	42	stroke	10	18	0.68	stick	34 (19)

* FMA-LE, Fugl-Meyer Assessment 0–34 (a higher score indicates better function)

** PASE, Physical activity scale for the elderly

*** Percentage of PASE age and sex matched reference values, in cohorts 40–79 years [33]

### Views and feelings about PA

#### Before stroke or ABI

When discussing PA in general, all participants expressed a positive attitude; PA was associated with good health and well-being.

It’s important to move your body to keep it going, your muscles and heart and fitness… simply to be healthy.

(Participant 10)

However, personal experiences of PA before stroke or ABI differed greatly between participants. Three persons stressed that PA had been an important part of their lives, which they now greatly missed. They had been engaged in activities like running, floorball, tennis and strength training. Others reported to be more or less novices in terms of physical exercise. Some had exercised over a period in life, and then quit because of physical impairments, lack of interest or time. All but one participant defined walking short distances, to the shop or bus station, as equivalent to exercise.

I’ve always exercised a lot… I played tennis at an elite level previously. I guess physical activity gives you mental strength.

(Participant 4)

I’m completely unexperienced with exercising, because I’ve never done it…and now I’m getting to start exercising here in the gym.

(Participant 3)

#### After stroke or ABI

PA was described as necessary, in order to rehabilitate and avoid a recurrent cardiovascular event. At the same time participants viewed PA as something demanding and not enjoyable—engaging in PA was more or less described as a necessary evil.

No, I’m not particularly motivated to exercise, but it’s gotten better since I started here (at the rehabilitation center). Because now I have to get out and about and go somewhere and move around and it feels good to be made to get out and about, and to do something like that (exercise).

(Participant 1)

I went to the gym even though it was hard, it wasn’t “great, how nice I get to go and exercise”, but I felt a sense of obligation to go.

(Participant 9)

Participants reported that they didn´t engage in PA apart from their program at the rehabilitation center. Most of them walked rather short distances; between 100 meters to 2–3 kilometers per week were reported.

I go supermarket shopping and then I go and have a lie down, because I feel that tiredness…

(Participant 5)

Well, it isn’t so terrible, I sit inside (at home) for the most part. But I walk to the tram stop nearby and it takes three minutes to go one way. Like there and back. And sometimes I go to the shops nearby here and they’re also really close.

(Participant 3)

Several participants expressed a vision of how they wanted to exercise in the future. These visions were combined with frustration over rehabilitation progress that was considered too slow.

Because I’ve exercised before. So I know how good it is and how much better it can be. But now it sure is hard work, but I can see how it can be, how it will be better. That’s the important thing.

(Participant 2)

### Perceptions of walking after stroke or ABI

Participants felt that they couldn´t walk in the same way as before. Their walking speed had slowed down, it was hard to walk straight, and they couldn´t walk for very long or had to stop frequently to catch their breath. Several were afraid of falling and some experienced problems with coordination and muscle weakness. These problems could lead to a leg or an arm sometimes being “left behind” when walking.

I move my feet a little differently every other step, but I’m used to that. And sometimes if I’m walking beside someone I can walk into them all of a sudden…And sometimes my feet do this (shows a crooked foot), and I’ve done that recently.

(Participant 6)

### Environmental factors disturbed walking

Participants discussed that too much input from their surroundings could be very disturbing and make walking even more difficult. Input that was considered disturbing could be auditory, visual or potentially harmful (traffic). Walking accompanied with too much input led to excessive problems with balance and fatigue, which also made these circumstances stressful and sometimes scary. Participants reported that they had to concentrate harder to walk straight and at a steady pace.

So, you are even more unsure of your balance in this kind of weather, when you’ve got this going on…because it’s almost twice as hard when I have a balance problem too. I have never actually fallen over. But you think about it a lot, yeah?

(Participant 5)

Bad weather was also described as an inconvenient factor that enhanced the risk of falling or losing balance. Several participants avoided walking if there was a risk of slipping, when it was raining or if snow lay on the ground.

I actually have a big problem with it (external input), for example if I’m out and walking with someone who is talking a lot, I get really tired…I have to concentrate on walking, I can’t have a lot of other things happening around me, I’m very sensitive to it.

(Participant 10)

Some participants mentioned that ground that was flat and even to walk on was important for them. Difficult terrain, for example walking in nature or climbing stairs was mentioned as especially troublesome.

It’s hard work…a thing like walking up-stairs for example, sometimes make me breathless…. but I barely dare (to walk outside) in this kind of weather because if I fall then I can’t catch myself, I would definitely be beaten to a pulp.

(Participant 6)

### Perceived impairments from the stroke or ABI affected PA engagement

Participants explained how and why they didn´t feel motivated or hadn´t enough energy to walk or engage in PA. A main issue that was brought up was fatigue and related problems such as lethargy, depression and a lack of initiative. Several participants reported feeling exhausted at times, they couldn´t manage to go out and they sometimes had to force themselves to get up from the couch. Problems with coordination and walking were, in some cases, related to impaired vision or diplopia.

I still go and lie down occasionally because of the tiredness. Such incredible tiredness… If I am tired then maybe I’m more likely to fall over if you know what I mean…

(Participant 8)

It’s hard to describe but a lot of it is mental… you’re always aware that you’ll never be fully well, that you are not the same (as before the stroke).

(Participant 5)

### Social and professional support facilitated engagement in PA and walking

Experiencing progress, for example; increased endurance, reduced body weight and stronger muscles, were considered high motivators for exercise. Social support and its meaning were discussed in different aspects. Having someone who accompanies you or who pushes you to be physically active, or having an appointment at the rehabilitation center were important for most participants. Meeting others in the same situation gave them hope, motivation and more realistic expectations. The rehabilitation team at the hospital was highly praised and participants were conscious of and expressed a fear of what would happen when they had to end their rehabilitation program. Several expressed that they might not exercise at all when this social and professional support had been ended.

Being in company. If you decide that you are going out with someone, you go for a longer time… It’s important that someone comes to get me out, and say that we are going out for walk now!

(Participant 4)

Participants stressed a need to feel secure during the actual exercise. They wanted advice about how to exercise in a safe way. A few also wanted someone to go with them because they were afraid of falling. Participants wanted to perform an exercise that they personally enjoyed and they wanted to feel well after having exercised.

You get a lot of advice about what to do and how you can exercise at home, and realize that it isn’t dangerous, that you have to put some effort in.

(Participant 9)

### PA and learnt strategies improved walking

Participants described how PA had improved their walking. Their ability to walk outside and climbing stairs was said to have improved through enhanced muscle strength and better balance. Several participants stated that they could now walk steadily for a longer period of time and at a higher speed thanks to an enhanced physical fitness.

It was easier to get to the tram a couple of days ago because I’d exercised for half an hour on the treadmill. So I could tell that I was getting results.

(Participant 3)

That’s what I’m doing now. Concentrating on a spot and just breathing. So it can seem as though it’s entirely quiet on the bus. And even when I’m walking, it’s easier…I can screen it out a little…when I do different activities, they say to me to sit down for ten minutes and just breathe, you just sitting and relax.

(Participant 1)

Participants discussed the importance of learnt strategies about how to cope better in everyday life that they had received through consultation with a psychologist. Several stressed that they had received individualized cognitive training that seemed tailor-made for their specific problems. Strategies mentioned were; how to breathe when stressed or tired, how to avoid exhaustion, blocking out disturbing input (as mentioned above) and how to interpret different signals from their bodies.

I listen more to my body now, and I’m not used to that. Now I immediately think, ‘listen to your body’, and stop straight away.

(Participant 2)

## Discussion

This study explored perceptions of walking and PA in people with stroke or ABI with similar symptoms, as well as experiences of PA before and after stroke or ABI. A majority of participants were rather physically inactive, as shown by their PASE-scores [33] (see Table 1). This is confirmed by existing literature, which showed that PA participation among persons with stroke is normally lower compared with a general population [36, 37], and that persons with stroke spend more time sitting and less time in activity than age-matched peers [9]. Most of the participants did not distinguish between general physical activity and planned exercise, these terms were used interchangeably. The lack of clear definitions and the use of mixed concepts for exercise and PA is a confirmed dilemma in previous qualitative research that aim to investigate these matters [10].

An interesting and somewhat concerning finding of this study was that while all participants considered PA to be very important to obtain good health, walking or PA was simultaneously associated with different negative feelings and experiences for them. Regardless of participants’ exercising habits pre-stroke, PA was defined as difficult and joyless after stroke. It is worth noting that participants who reported that PA had been an important part of their lives pre-stroke did not enjoy PA of any kind and had to use motivational strategies—or even be more or less forced by others to engage in PA after stroke. These results are also alarming because participants in our study were a lot younger, with a mean age of 50 years, compared to that of a general stroke population in Sweden, mean 75 years [38], and might be expected to be more motivated to engage in PA in order to regain functioning in everyday life and get back to work [39].

Previous studies have reported similar results, since physical and cognitive impairments after stroke are well known to disturb exercise performance and to make exercise more difficult [10, 20, 23, 25, 40]. That persons with stroke are aware of the relationship between PA and good physical health have also been confirmed previously [10, 20], even though some studies [25, 40, 41] reported the opposite. For example one study from Chicago (USA) showed that 40% of participants didn´t believe that exercise would improve their condition [25].

Participants described the ability to walk as highly important in their everyday lives. Walking straight, at a steady pace, without fear of falling were desirable skills. It has been confirmed that improving walking ability is the most reported rehabilitation goal for persons with stroke [15, 16]. In a study from Ireland, persons with stroke were asked which component of their physiotherapy program they liked the most; walking was the most frequent response, followed by lower-extremity exercises [42]. Participants mentioned hardships in relation to walking, for example; difficulties with walking straight, low endurance when walking and a perception of walking too slowly.

There was also a concern about not walking “like everyone else”, which might have been a fear of being looked upon as someone with a disability. This finding was in accordance with a study from the USA, where participants wanted to walk with greater ease and better coordination so that they wouldn´t look like someone with a disability [17].

Participants communicated numerous motivators of and barriers to PA and walking. While the results of our study also included perceptions of walking, mentioned motivators and barriers were similar to previous research that investigated perceptions of PA more generally [20, 22, 23, 25]. Commonly reported internal barriers from the current study, in line with previous research [20, 23, 25, 40] were; fatigue, fear of falling or getting hurt in traffic, lack of motivation and depression. According to a study from Oregon, USA [43], persons with a recent brain injury reported; insufficient endurance (29%), feeling self-conscious in a fitness center (25%) and not enough time to engage in PA (21%) as the main personal barriers.

Loneliness and boredom were additional internal barriers reported in previous research [10, 20]. The lack of these factors in the current study might simply be because our participants had had their stroke or injury more recently, and were still patients being cared for at the rehab clinic, and that feelings of boredom and loneliness might take longer to experience [44]. In discussion about how to handle mentioned barriers, participants brought forth that they needed a lot of social support from family, friends, or healthcare professionals. Having someone to walk with or an appointment was important. The importance of such social support and motivation was the most reported internal motivator, which is also in line with previous research [26, 45, 46].

In addition, participants feared that they wouldn´t be able to keep up with physical exercise when their rehabilitation program ended. This awareness was confirmed by a study from Baltimore [26], where persons with stroke admitted that they wouldn´t come to the rehabilitation program to exercise without external support. Another study reported that participants in their study were typically disappointed because professional services ended too quickly [45].

Apart from being instructed on how to perform exercise adapted to one´s functional level, several participants valued learnt strategies to overcome cognitive impairments and fatigue highly, e.g., how to breath, or block out disturbing input and when to rest in order to avoid exhaustion in everyday life. The experienced value of learnt strategies to handle fatigue has not been acknowledged in research before. Our results showed not only that these strategies were described as crucial tools for handling everyday tasks, but also that they were very important to keep energy levels up throughout the day. From our understandings, strategies that aim to facilitate daily life would probably be important to most persons who suffer from post-stroke fatigue.

Reported external barriers were mostly related to walking, for example bad weather, uneven ground, lack of company or a noisy or too busy surroundings. These factors have, as far as we know, not been reported previously. As the current study was carried out in a geographical area (Sweden) where weather conditions can be harsh and difficult for outdoor activities for anyone, persons with disabilities may be even more dependent on weather conditions that are good enough. This result highlights the need of access to indoor exercise facilities for persons with disabilities who live in geographical areas with recurrent harsh weather conditions.

Other studies of perceptions of PA have also identified; change in work or life status, lack of economical means to attend rehabilitation programs, too few places available to exercise or poor opportunities of transportation [10, 20, 25, 43]. None of these external factors were mentioned in our study. Participants in the current study were assessed at a relatively early stage (2–10 months post injury), still on sick-leave and attending tax-funded rehabilitation with access to subsidized transportation. It is possible that these circumstances together meant that they hadn´t yet resumed their daily life in order to encounter barriers associated with a later stage post-injury [47]. Another explanation could be that all participants were referred to an ongoing rehabilitation program that they liked, and therefore didn´t feel a need for, or had energy to, carry out additional physical exercise at time of the interview.

### Strengths and limitations

We aimed for a range in participants’ age, sex and functional status. The study group was considered representative for the rehabilitation clinic regarding sex distribution. A variation in disability status was reflected by a dispersion of the participants’ walking speeds, which were low in comparison with a reference population [28, 48].

Since the participants had a lower mean age than a general stroke population, and a quite good communicative ability, it is possible that the results are less representative for the whole stroke population. Another limitation is that the study did not include a measure of cognitive function. That both persons with stroke and ABI were included makes interpretation for a specific patient group difficult, however this mix of diagnoses was considered as fairly representative for the population at the rehabilitation unit.

The content of the result was rich in relation to the research questions and very little new information emerged after about ten interviews. Therefore, we decided that the current number of participants were sufficient for this study.

The qualitative design allowed an open mind regarding the research questions; hence the result was not limited by the content of a questionnaire. The method also enabled issues to be examined in detail and in depth. For example, as part of the aim was to examine perceptions about PA, the interview process allowed us to investigate what participants meant when they spoke about PA. Most participants defined PA as equivalent to walking, and by knowing that, we made sure to use the same definition.

However, even though the result of the current study replicated much from previous research investigating the same area, participants in the present study had a lower mean age and were still undergoing rehabilitation. Therefore, our results might not be valid for persons post-stroke or ABI of an older age or in later stages of rehabilitation. The findings are important because younger participants can be assumed to live with disabilities for a longer time. As a secondary prevention PA is important in all ages. However, vocational activities and family life requires a quite high endurance. We therefore assume that interventions promoting PA would be of outmost importance in rehabilitation for people of working age.

## Conclusions

In the rehabilitation phase, younger persons post-stroke or ABI, communicated a need for both internal and external motivators in combination with a limitation of barriers in order to engage in PA or walking. Therefore, when forming rehabilitation interventions, professionals in the field of stroke and ABI rehabilitation should take the perceptions and concerns held by persons with these conditions into account. With increased knowledge about barriers and motivators to PA it should be possible to enhance motivators and to decrease barriers, so that this group won’t fear or avoid PA.

This study showed clearly that persons with stroke or ABI needed rehabilitation that included learning strategies to cope with fatigue and an overload of input in everyday life. Meeting these concerns seems crucial in order to motivate this group to engage in PA. Another challenge for physical therapists and other healthcare professionals might be to mediate personal motivation and commitment during the rehabilitation process. Involvement of families can also be important to meet the need for additional external motivation. Future research should be focused on how to form rehabilitation interventions that are based on concerns about PA held by these groups, and in the next stage evaluate the effectiveness of this approach.

## Supporting information

S1 File Interview guide—Physical activity. Interview guide—Physical activity.
(PDF)
