# Supplementary material for: Perceptions of physical activity and walking in an early stage after stroke or acquired brain injury
Source: PLoS One. 2017 Mar 8;12(3):e0173463. doi: 10.1371/journal.pone.0173463 (PMC5342245; doi:10.1371/journal.pone.0173463)
Supplement: S1 File — Interview guide—Physical activity. (PDF) [file pone.0173463.s001.pdf]

Interview guide

How would you describe your general view of Physical Activity?

Before the stroke/brain injury:

Would you say that Physical Activity was an important/non important part of your life?

How often did you engage in Physical Activities?

What did you engage in, and what did you think about it?

After the stroke/brain injury:

How do you feel about Physical Activities today?

Approximately, how often do you engage in Physical Activities?

How do you feel about that?

What do you think about the rehabilitation exercise?

Do you feel uncomfortable when you do the exercise? (Pain, dizziness or tiredness for example)

How do you feel about walking now? (Pain, dizziness or tiredness for example)

Approximately, how much do you walk during a normal day?

Are there factors that can make you walk less during a normal day? (For example; mental, physical barriers, or something else)

Are there factors that can encourage you to walk more during a normal day? (For example; motivation, a good night’s sleep or something else)

Do you engage in any form of physical exercise to improve your walking? (Speed, balance or endurance)

Do you engage in any form of physical exercise to improve your strength or endurance?
